# Feasibility of a kinect-based system in assessing physical function of the elderly for home-based care

**DOI:** 10.1186/s12877-023-04179-4

**Published:** 2023-08-16

**Authors:** Xin-Ting Liu, Mohammad Nikkhoo, Lizhen Wang, Carl PC Chen, Hung-Bin Chen, Chih-Jui Chen, Chih-Hsiu Cheng

**Affiliations:** 1 School of Physical Therapy and Graduate Institute of Rehabilitation Science, College of Medicine, Chang Gung University, No.259, Wen-Hwa 1st Rd, Kweishan, Taoyuan, Taiwan, R.O.C.; 2 Bone and Joint Research Center, Chang Gung Memorial Hospital, Linkou, Taiwan, R.O.C.; 3 Department of Biomedical Engineering, Science and Research Branch, Islamic Azad University, Tehran, Iran; 4 School of Biological Science and Medical Engineering, Beihang University, Beijing, China; 5 Department of Physical Medicine & Rehabilitation, Chang Gung Memorial Hospital at Linkou and College of Medicine, Chang Gung University, Taoyuan, Taiwan, R.O.C.; 6 LongGood Meditech Ltd, Taipei, Taiwan, R.O.C.

**Keywords:** Telemedicine, Kinect, Physical fitness, Motion analysis, Reproducibility of results, Validation studies

## Abstract

**Background:**

With concerns about accurate diagnosis through telehealth, the Kinect sensor offers a reliable solution for movement analysis. However, there is a lack of practical research investigating the suitability of a Kinect-based system as a functional fitness assessment tool in homecare settings. Hence, the objective of this study was to evaluate the feasibility of using a Kinect-based system to assess physical function changes in the elderly.

**Methods:**

The study consisted of two phases. Phase one involved 35 young healthy adults, evaluating the reliability and validity of a Kinect-based fitness evaluation compared to traditional physical examination using the intraclass correlation coefficient (ICC). Phase two involved 665 elderly subjects, examining the correlation between the Kinect-based fitness evaluation and physical examination through Pearson’s correlation coefficients. A Kinect sensor (Microsoft Xbox One Kinect V2) with customized software was employed to capture and compute the movement of joint centers. Both groups performed seven functional assessments simultaneously monitored by a physical therapist and the Kinect system. System usability and user satisfaction were assessed using the System Usability Scale (SUS) and Questionnaire for User Interface Satisfaction (QUIS), respectively.

**Results:**

Kinect-based system showed overall moderate to excellent within-day reliability (ICC = 0.633-1.0) and between-day reliability (ICC = 0.686-1.0). The overall agreement between the two devices was highly correlated (r ≧ 0.7) for all functional assessment tests in young healthy adults. The Kinect-based system also showed a high correlation with physical examination for the functional assessments (r = 0.858–0.988) except functional reach (r = 0.484) and walking speed(r = 0.493). The users’ satisfaction with the system was excellent (SUS score = 84.4 ± 18.5; QUIS score = 6.5–6.7).

**Conclusions:**

The reliability and validity of Kinect for assessing functional performance are generally favorable. Nonetheless, caution is advised when employing Kinect for tasks involving depth changes, such as functional reach and walking speed tests for their moderate validity. However, Kinect’s fundamental motion detection capabilities demonstrate its potential for future applications in telerehabilitation in different healthcare settings.

**Supplementary Information:**

The online version contains supplementary material available at 10.1186/s12877-023-04179-4.

## Background

The global populaton of individuals aged 65 years or older is growing at a rate of approximately 9 million people per year. By 2050, this population is projected to double and reach nearly 2 billion, indicating a significant demographic shift toward an aging society [1]. It leads to increasing cost pressures through rises in health and social care expenses. Additionally, the COVID-19 pandemic has necessitated substantial modifications in healthcare delivery, leading to the widespread adoption of telehealth [2, 3]. However, the elderly population often requires care within the comfort and safety of their own homes, raising concerns about the accuracy of telehealth diagnoses. Clinicians have voiced concerns, deeming telehealth as substandard compared to in-person or face-to-face care [4]. Hence, it is essential to develop new strategies for primary health care, including nursing homes and home care services [5]. To facilitate the role of home care and promote independence among elderly individuals, abundant innovative solutions and advanced technologies have been introduced within the healthcare system. These initiatives aim to enhance the quality of care provided at home and foster a community-based approach for the elderly population [6].

Responding to the problem of insufficient manpower for elderly care and the problem of increasing social costs, “smart home care” and “technological health management” could be a noble solution with the increase in the elderly’s acceptance rate of technology and the rapid growth of uses of digital devices [7, 8]. These health management techniques offer significant benefits for the physiological and cognitive function of elderly patients [9], such as the management of aging-related chronic diseases by monitoring vital signs [10, 11], psychological conditions, controlling depression, and reducing the post-discharge problems [12]. Although abundant studies have been focused on the application of technological health management, limited researches make use of smart health strategies as a fashion in home diagnosis or predicting diseases or disability of the elderly population [6, 13].

Quantifying the physiological capacity of the elderly and assessing their functional fitness performance is essential for enhancing their quality of life [14]. By objectively measuring their physical capabilities, healthcare providers can gain valuable insights into their overall health status, identify areas for improvement, and tailor interventions to promote better well-being and independence [15]. Inspection of agility and dynamic balance are two main indexes to achieve early identification of fall risk in the elderly [16] which can be used in addition to quantifying the body strength and mobility for evaluating the physiological capacity [15]. Through functional fitness performance, it can provide a personalized program to improve the case’s lack of ability and assess the effectiveness of the intervention based on systematic follow-up. The low motivation and challenges faced by elderly individuals in attending physical training sessions at clinics often result in insufficient exercise [17]. However, with the integration of scientific and technological health management, traditional physical fitness testing can be optimized. These advancements offer the potential to overcome barriers and provide more accessible and convenient solutions for assessing and improving the physical fitness of the elderly population [18]. Application of new technologies and facilities to increase the training motivations in elderly and consequently improve their functional performances or disabilities could be crucial for adopting health management strategies [19, 20].

It is common to utilize sensors or wearable devices for functional fitness examinations based on the evaluation of the impairment in physical activities [21], and abundant research has utilized different techniques and approaches for this purpose. While wearable sensors offer convenience and are commonly used in research laboratories, the quality of collected signals relies on correct positioning to minimize potential noise [22]. This dependency on accurate sensor placement poses challenges for the widespread application of wearable sensors in home-care systems. Ensuring consistent and reliable signal quality in a home-care setting may require additional considerations such as user education, proper sensor placement guidance, and potential advancements in sensor technology that mitigate noise and improve signal accuracy [23]. Conversely, optical motion capture cameras could be used as an accepted method of patient motion analysis [24, 25]. Therefore, the Microsoft Kinect sensor can be utilized as a robust, marker-free, low-cost, and reliable device that can record proximity and depth data in real-time [26–29].

Previous research studies have established the reliability and validity of utilizing Kinect technology for the analysis and detection of balance, gait, and posture control [30–32]. By leveraging Kinect’s capabilities, healthcare professionals can obtain accurate and objective measurements to evaluate the functional movements of individuals, leading to improved diagnoses, interventions, and overall healthcare outcomes. Hence, the Kinect could be used as an encouraging technology for providing reliable solutions for patient movement analysis in home-care systems [27, 33]. However, there is a lack of a practical study to comprehensively investigate the feasibility of developing a functional fitness assessment system using Kinect sensors, which can be beneficial for developing homecare strategies. Hence, the main objective of this study was to evaluate the feasibility of this technique for the assessment of changes in physical function in elderly.

## Methods

### Recruitment

#### Phase 1: Assessment of young subjects

Thirty-five young healthy volunteers (25.0 ± 5.7 years old, 168.3 ± 8.4 cm, and 62.5 ± 11.9 kg), including 19 males (25.7 ± 5.6 years old, 174.5 ± 4.8 cm, and 68.7 ± 12.2 kg), 16 females (24.3 ± 5.9 years old, 160.9 ± 5.2 cm, and 55.0 ± 5.7 kg) were recruited in the first phase of this study to evaluate the reliability and validity of a Kinect based fitness evaluation and traditional physical examination. The sample size of the experimental tests using a power test of 95% and a significance level of 0.05, was at least 34 participants. Accordingly, the sample size condition was fulfilled in the first phase of this study by including 35 young healthy volunteers. The healthy volunteers participated in the test-retest study at the Movement Science and Assistive Technology Laboratory of Chang Gung University for two separate testing sessions approximately seven days apart. All participants met the following inclusion criteria: (1) age 18 or over, (2) participants can easily operate with both hands (3) participants can walk independently for at least 10 m. On the other hand, the participants were excluded if any of the following conditions occurred: (1) adopting cardiac pacemakers or obvious arrhythmia, infection, cancer, and hematological and other internal diseases (2) acute visual disturbance or reading disorder that may make difficulty for reading the instructions on the screen.

#### Phase 2: Assessment of elderly subjects

Six hundred and sixty-five elderly subjects (70.0 ± 5.7 years old, 160.2 ± 8.1 cm, and 60.5 ± 10.2 kg), including 234 males (70.7 ± 5.5 years old, 167.3 ± 6.5 cm, and 67.2 ± 10.0 kg), 431 females (69.6 ± 5.8 years old, 156.3 ± 6.1 cm, and 56.9 ± 8.2 kg) were recruited in the second phase of this study to examine the cross-mode similarity between the Kinect based fitness evaluation model and the physical examination approach. The second phase of this study fulfilled the sample size requirement, as a minimum of 567 participants was determined based on a power test of 95% and a significance level of 0.05. The elderly volunteers participated in this cross-sectional study at the National Taiwan University Hospital and Changhua Christian Hospital in Taiwan as well as the National Research Center for Rehabilitation Technical Aids in China from 2020 to 2022. The inclusion criteria were as follows: (1) age 55 or over, (2) participant can easily operate with both hands (3) participants can walk independently for at least 10 m using or not using assistive devices. The exclusion criteria were the same as the recruitment criteria for healthy volunteers in the first phase.

Signed informed consents were acquired from all subjects in both experimental phases prior to their enrolment in this clinical protocol, which was approved by the university ethics committee (Chang Gung Memorial Hospital, Approval number: 201900410B0A3), and all subjects provided informed consent. Adequate explanations about the experimental test procedure were given to all subjects prior to performing the experiments.

### Experimental test procedure

The Kinect sensor (Microsoft Xbox One Kinect V2, Microsoft Corporation, Redmond, WA, USA) equipped with customized software was used to capture and calculate the movement of joint centers when performing the functional evaluation. The signal obtained by Kinect V2 was collected using a Mini PC (Intel Core i5-8259U @ 2.30 GHz, 16 GB of RAM, and Intel Iris Plus Graphics 655). Customized software (LongGood MediTech Ltd. Taipei, Taiwan) was employed to calculate data related to various functional actions. Furthermore, the user interface of the customized software system was displayed on the screen. To obtain the ideal data, the recommendations for using the Kinect sensor were carefully considered (i.e., using tight clothes, no shiny black fabric or reflexes, no moving hair, and no sunlight).

Subjects performed the following seven functional assessment tests, simultaneously monitored by a professional physical therapist and the Kinect system, including: (1) one-leg stance (OLS); (2) functional reach (FR); (3) sit-to-stand (STS); (4) timed-up to go (TUG); (5) arm curl (AC); and (6) two-minute step test (TMST). These physical tests were selected to evaluate the ability to keep balance, the muscular strength of the upper and lower body, agility, and aerobic endurance, respectively [34–36]. In addition, gait analysis was performed to assess the subjects’ mobility based on their walking speed. The details of experimental test procedures are described in Table 1. To avoid fatigue of the extremities, the sequence of the tests was performed from test No. 1 to 6 and two-minute rests among the tests were considered. All subjects could easily follow the guidance from the screen to perform the aforementioned experimental procedure.

**Table 1:** Movement protocol from the movement tasks

Movement	Ability	Test Procedure
One Leg Stance	Balance	The users stand on their dominated leg without assistive device, and keeping their arms by sides. The test is over after 45s has elapsed. When the stance foot shifts, or when the lifted foot collapsed, the test is over, too. The stance time will be recorded [37].
Functional Reach	Balance	The users stand in a neutral position and then raise the arm forward to shoulder height. As instructed, the user is then asked to reach forward as far as possible without moving the feet while keeping the arm horizontal. The distance of the tip of the middle finger between initial and the furthest positions was recorded [38].
Sit to Stand	Lower body strength	The users are asked to stand and sit as fast as possible repeatedly for 30 s with arms folded across chests. The number of repetitions is then recorded. Besides, only full stands and sits will be identified [39].
Timed-Up to Go	Agility and balance	A chair is firstly set 4 m away from the Kinect sensor. Users are instructed to stand from a seated position in a chair, walk at their fastest pace for 3 m, turn around, return to the chair and sit back down. Total time is then recorded [15].
Arm Curl	Upper body strength	The users begin with sitting on a chair without the arm rest. Dumbbell measuring 5 lbs and 8 lbs is applied for females and males respectively. The number of flexion-extension of the dominant elbow within 30 s are recorded [15].
2-Min Stepping	Aerobic endurance	Users are asked to raise each knee to a point midway between the patella and iliac crest. And they must alternately raise their knees as fast as possible for 2 min. The number of full steps is then recorded [15].
Gait Speed	Mobility	Users are asked to stand in a neutral position 4 m away from the Kinect sensor at the beginning. They are then instructed to walk forward at their usual pace. The gait speed is calculated and recorded.

**Fig. 1:**
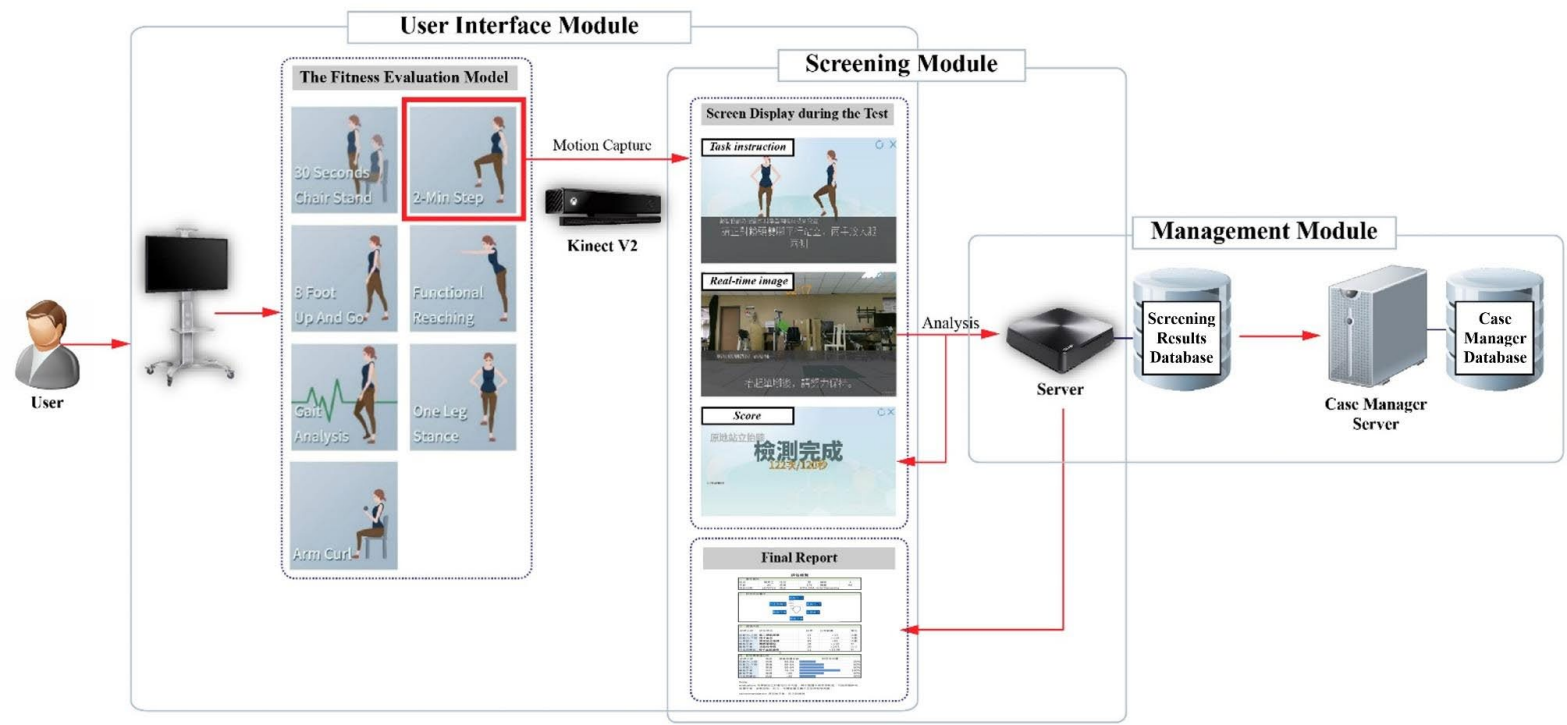
General overview of the architecture of the developed system

Prior to starting the tests, the screen showed audio guidance to participants with animations and subtitles to explain how to perform the experimental procedure step by step. A five-second countdown was predicted to get the participants ready to perform the requested functions. When the countdown ends, Kinect V2 begins to capture the movement of joint centers and the actual images displayed on the screen for better visual feedback at the same time. At the end of each test, the screen shows the test results and the system can output a final report. In the meantime, an expert examiner manually evaluated the functional tests and measured the gait speed using a chronograph. Figure 1 shows the schematic procedure of the developed system.

### Experimental test evaluations and data analysis

These movements were captured relative to the Kinect V2 camera located directly in front of the subjects. Three-dimensional motion parameters were acquired from the data of 25 joint points identified by the skeleton tracking system at 30 Hz and the movement of joint centers was calculated using the customized software (LongGood MediTech Ltd. Taipei, Taiwan) [40]. In the first phase, reliability and validity analyses of the developed fitness evaluation model were evaluated for young subjects. Each experimental test was repeated two times on the same day to assess the “within-day reliability” and participants attended two separate testing sessions approximately seven days apart to evaluate the “between-day reliability”. In addition, the comparisons of the fitness evaluation using the Kinect-based system and the traditional physical examination system were evaluated using criterion-related validity.

**Fig. 2:**
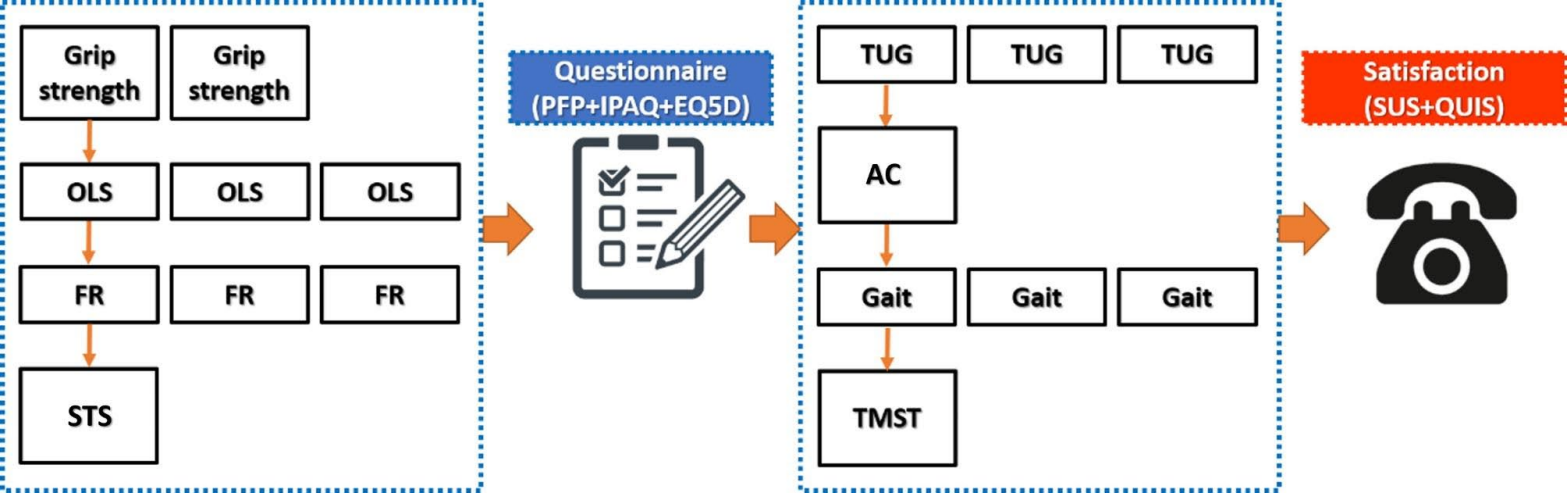
Schematic study protocol (OSL: one-leg stance; FR: functional reach; STS: sit-to-stand; TUG: timed-up to go; AC: arm curl; and TMST: two-minute step test)

In the second phase, the cross-mode similarity between the Kinect-based and physical examination techniques was evaluated for elderly subjects who were our target group. The System Usability Scale (SUS) was utilized to inspect the acceptability of the system. The SUS included 10 questions about several aspects of subjects’ feelings, such as ease of use, satisfaction, importance, happiness, and usefulness (Appendix 1). The SUS items included 5 forward questions (question 1,3,5,7,9) and 5 reverse questions (question 2,4,6,8,10) [41, 42]. Each item can be scored on a 5-point Likert scale from 1 (strongly dissatisfied) to 5 (strongly satisfied). For overall satisfaction, the Questionnaire for User Interface Satisfaction (QUIS) was used. The prepared questionnaire contained four aspects, including (1) overall experience with the system, (2) quality of the display contents, (3) system messages readability, and (4) ease of learning how to use the system based on the system guidelines. The QUIS included 16 questions (Appendix 2) which can be scored on a 7-point Likert scale from 1 (strongly dissatisfied) to 7 (strongly satisfied). We separated the two blocks of functional assessment tests by the questionnaire assessments to minimize the fatigue effects (Fig. 2).

### Statistical analysis

All statistical analyses were performed in SPSS (SPSS Inc., Chicago, IL, USA). The reliability (within-day and between-day) of the Kinect-based system was analyzed by the intraclass correlation coefficient (ICC) with a 95% confidence interval (CI) using a two-way mixed effect model based on single ratings and absolute agreement. The achieved results can be classified into four groups [43] (i.e., “poor” for ICC < 0.5, “moderate” for 0.5 ≦ ICC < 0.75, “good” for 0.75 ≦ ICC < 0.9, and “excellent” for ICC ≧ 0.9). The 95% CI for the limits of agreement (LOA) was evaluated to check the concurrent validity of the Kinect-based system compared to the physical examination. Pearson’s correlation coefficients were calculated the interclass correlation (r) to evaluate the consistency of the proposed techniques for different functional assessment tests. The level of relationship between the two techniques can be categorized into four groups (i.e., “modestly correlated” for r < 0.39, “moderately correlated” for 0.4 < r < 0.69, “highly correlated” for 0.7 < ICC < 0.99, and “perfectly correlated” for r = 1). Further, a non-parametric methodology was implemented for the Bland-Altman analyses. Agreement between the two techniques was plotted graphically by Bland–Altman plots for different functional assessment tests. The 95% limits of agreement were calculated as mean difference ± 1.96 standard deviation (SD) of the differences. Finally, descriptive statistical analyses of the outcome measurements from SUS and QUIS were performed.

## Results

Means and standard deviations of outcome measures during all functional assessment tests to investigate the validity and reliability of results for young healthy volunteers are shown in Table 2. The ICC values of “within-day” and “between-day” reliability ranged from 0.633 to 1.0 and 0.686 to 1.0 for the Kinect-based system and physical examination, respectively (Table 2). Moderate to excellent reliability was found for both “within-day” and “between-day” for all tests (i.e., “good” to “excellent” reliability for OLS, STS, AC, and TMST, and “moderate” reliability for FR, TUG, and GS). The overall agreement between the two devices was highly correlated (r ≧ 0.7) for all functional assessment tests in young healthy volunteers (Table 3).

**Table 2:** Evaluation of the reliability for the Kinect-based fitness evaluation model and physical examination (PE).

Movement	Within Day 1			Within Day 2			Between Days		
	Trial 1	Trial 2	ICC	Trial 1	Trial 2	ICC	Day 1	Day 2	ICC
**OLS(s)**									
**PE**	45.0 ± 0.0	45.0 ± 0.0	1.0	44.8 ± 1.4	45.0 ± 0.0	1.0	45.0 ± 0.0	44.9 ± 1.0	1.0
**Kinect**	45.0 ± 0.0	45.0 ± 0.0	1.0	44.8 ± 0.9	45.0 ± 0.0	1.0	45.0 ± 0.0	44.9 ± 0.7	1.0
**FR (cm)**									
**PE**	36.5 ± 4.9	37.2 ± 5.9	0.895	37.2 ± 5.6	38.5 ± 5.8	0.939	36.9 ± 0.5	37.8 ± 0.9	0.889
**Kinect**	36.4 ± 5.6	37.6 ± 5.5	0.735	38.1 ± 5.9	37.1 ± 5.8	0.809	37.0 ± 0.9	37.6 ± 0.7	0.777
**STS (times)**									
**PE**	22.2 ± 4.3	25.0 ± 5.3	0.903	24.9 ± 5.3	26.1 ± 5.1	0.945	23.6 ± 2.0	25.5 ± 0.9	0.815
**Kinect**	22.1 ± 4.5	24.9 ± 5.3	0.901	24.7 ± 5.5	26.0 ± 5.2	0.942	23.5 ± 2.0	25.4 ± 0.9	0.797
**TUG (s)**									
**PE**	5.3 ± 0.7	5.1 ± 0.7	0.927	5.1 ± 0.7	5.0 ± 0.6	0.910	5.2 ± 0.2	5.0 ± 0.1	0.799
**Kinect**	5.2 ± 1.2	5.1 ± 1.1	0.933	5.2 ± 1.1	4.9 ± 0.9	0.679	5.2 ± 0.1	5.0 ± 0.2	0.764
**AC (times)**									
**PE**	21.7 ± 5.2	23.2 ± 5.5	0.961	23.5 ± 4.1	24.2 ± 4.7	0.932	22.5 ± 0.2	23.9 ± 0.5	0.902
**Kinect**	21.6 ± 5.2	23.1 ± 5.5	0.960	23.4 ± 4.1	24.2 ± 4.7	0.932	22.3 ± 1.0	23.8 ± 0.6	0.903
**GS (m/s)**									
**PE**	1.1 ± 0.2	1.1 ± 0.2	0.810	1.2 ± 0.2	1.2 ± 0.2	0.854	1.1 ± 0.0	1.2 ± 0.0	0.686
**Kinect**	1.0 ± 0.2	1.1 ± 0.2	0.873	1.1 ± 0.2	1.1 ± 0.2	0.795	1.1 ± 0.0	1.1 ± 0.0	0.633
**TMST (steps)**									
**PE**	309.0 ± 75.1	326.3 ± 76.3	0.929	323.5 ± 73.7	335.9 ± 65.5	0.849	317.7 ± 12.2	329.7 ± 8.7	0.910
**Kinect**	304.6 ± 76.0	319.4 ± 79.5	0.937	321.0 ± 76.0	335.1 ± 65.0	0.852	312.0 ± 10.5	328.1 ± 10.0	0.874

In the second phase, the Kinect-based system and physical examination showed “highly correlated” for the OLS, STS, TUG, AC, and TMST (r values ranged from 0.858 to 0.988), and “moderately correlated” for the FR and GS (r values were 0.484 and 0.493, respectively) (Table 4).

**Table 3:** Evaluation of the validity for the Kinect-based fitness evaluation model and physical examination (PE).

	PE	Kinect	Correlation (r)
One Leg Stance (OLS) (sec)	44.9 ± 0.7	44.9 ± 0.5	1.000
Functional Reach (FR) (cm)	37.8 ± 5.5	37.6 ± 5.4	0.823
Sit to Stand (STS) (times)	25.5 ± 5.1	25.4 ± 5.2	0.998
Timed-Up to Go (TUG) (sec)	5.1 ± 0.6	5.1 ± 0.8	0.741
Arm Curl (AC) (times)	23.9 ± 4.2	23.8 ± 4.2	0.999
Gait Speed (GS) (m/s)	1.2 ± 0.2	1.1 ± 0.2	0.858
Two min Stepping (step)	329.7 ± 65.0	328.1 ± 66.0	0.998

**Table 4:** Cross-mode similarity between the Kinect-based fitness evaluation model and physical examination

	PE	Kinect	*r*	*P*
One Leg Stance (sec)	25.7 ± 16.1	26.2 ± 16.1	**0.988**	0.000*
Functional Reach (cm)	33.2 ± 5.3	33.3 ± 6.6	**0.484**	0.000*
Sit to Stand (times)	16.1 ± 4.2	15.8 ± 4.4	***0.948***	0.000*
Timed-Up to Go (sec)	7.1 ± 1.5	8.1 ± 2.1	***0.858***	0.000*
Arm Curl (times)	16.6 ± 4.8	16.5 ± 4.5	***0.931***	0.000*
2-min Stepping (step)	210.5 ± 39.0	207.7 ± 40.1	***0.940***	0.000*
Gait Speed (m/s)	1.2 ± 0.2	1.3 ± 0.3	***0.493***	0.000*

**Fig. 3:**
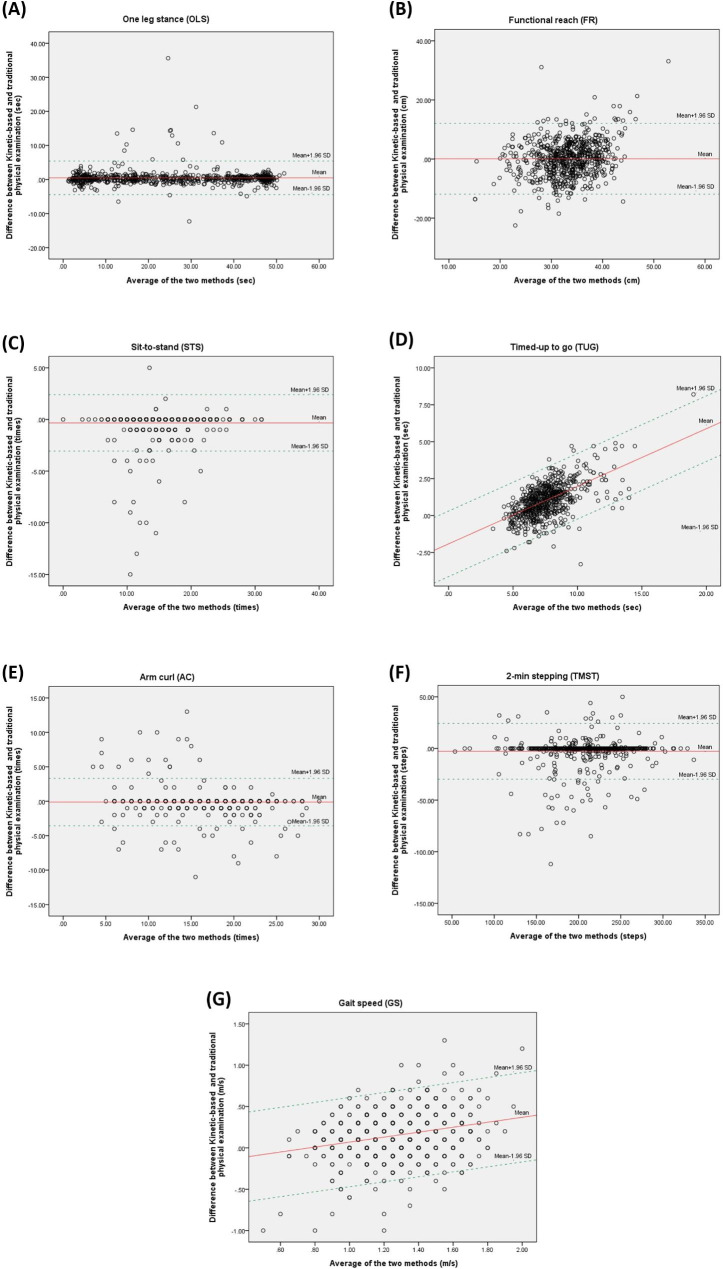
Bland–Altman plots for different functional assessment tests. Mean difference between Kinect-based and traditional physical examination: limits of agreements (± 1.96 ∗ standard deviation (SD))

Visual inspection of the Bland–Altman plots revealed a uniform variability for the majority of tests which was confirmed by low correlation coefficients between the mean values and the absolute difference from the Kinect-based system and physical examination technique (Fig. 3). In addition, the average calculated SUS score was 84.4 ± 18.5 which is rated as excellent for user satisfaction. Items No. 3 and 5 in forward questions and item No. 4 in reverse questions got the highest average scores. The average scores for each question in the QUIS evaluation technique ranged from 6.5 to 6.7.

## Discussion

The ability to evaluate the functional fitness of the elderly using a portable, noncontact, and easy-to-use system could provide research and clinical benefits. The current study aimed to assess the concurrent reliability and validity of a developed functional fitness system using the Kinect sensor compared to the traditional physical examination technique. The Kinect system is a feasible and practical motion-sensing device [27, 44] to extract skeletal movement data. The application of the Microsoft Kinect was formally validated to assess impairment and balance compared to the gold standard instruments such as wearable sensors and motion capture techniques [45–47]. The findings of this study highlight the substantial reliability and validity of the functional fitness system in both young and elderly populations. The strong correlation observed between the movements analyzed through this system and traditional physical examination methods suggests that the Kinect sensor holds substantial promise for integration into clinical screening programs. The selected functional tests employed in this study were deliberately designed to be simple and easily performed without the need for specialized equipment or utilities. Consequently, this technique has the potential to be readily implemented in patients’ homes or small clinics, making it a suitable approach for telerehabilitation initiatives. In addition to its practical applicability, the feedback survey conducted among elderly individuals who utilized this system revealed their positive experiences. Specifically, the system was praised for its excellent usability and interface legibility. These aspects contribute significantly to enhancing Information and Communication Technology (ICT) based health management systems. By providing an intuitive and user-friendly platform, this functional fitness system has the potential to contribute positively to the advancement and effectiveness of ICT-driven health management approaches. Given its reliability, validity, simplicity, and user-friendly nature, the functional fitness system, with the integration of the Kinect sensor, emerges as a valuable tool with the potential to enhance clinical screening, facilitate telerehabilitation in diverse settings, and improve the overall quality of ICT-based health management systems. Further research and implementation are warranted to fully explore the scope and benefits of this technology in broader healthcare contexts.

Compared to traditional health management strategies, taking advantage of the ICT and Internet of Things (IoT) can be a technology-based way to play up “personalized” and ”accessible” in homecare services. Numerous studies have provided evidence supporting the notion that these emerging technologies have the capacity to decrease the elderly’s reliance on family members or caregivers [48] while simultaneously improving their quality of life [49, 50]. Smart technologies can contribute to optimizing health management in medical institutions, executing accurate clinical decisions, and setting up customization. The progress of ICT in health management has the potential not only to facilitate the diagnosis and treatment to distant patients, tightening up the relationship between the seniors and their caregivers and medical workers but also to enable their family members to understand the elders’ physical conditions [6]. Although numerous research activities have been focused on the application of ICT for health management, limited researches make use of smart health strategies as a fashion in home diagnosis or predicting diseases or disability of the elderly population [6, 51]. Functional motion detection has proven to be effective in predicting disability and fall risk in the past. The findings of this study demonstrate the utilization of this system for remote monitoring of cases and systematic collection of data. The collected data is then sorted and stored in an organized manner. Furthermore, machine learning algorithms can be applied to calculate the severity of the case. The functional actions capture by the system correspond to the level of disability or overall health status.

A comparative investigation of the achieved results in the first and second phases of this study demonstrated a higher correlation coefficient (r) between Kinect and physical examination for young subjects, excluding the TUG test. Based on analyzing the motion patterns, we found that this subject may refer to the smaller values of joint angles and movement amplitudes for the elderly group, which can lead to errors in motion capturing using the Kinect system [52]. This is consonant with the fact that seniors change their activity strategies by reducing their movement range in exchange for physical stability and balance [53]. Similar results have been discussed in the literature [54] by assessing the changes in body centroid of the static and dynamic balance of the young and the elderly groups using Kinect, in which the validity of the elderly is slightly lower than that of the young subjects.

On the other hand, the correlation coefficient for FR and GS tasks significantly dropped to 0.484 and 0.493, respectively, which is classified as moderately correlated. Regarding the FR test, we recognize that the judgment of the developed software may be different from the standard measurements of the traditional physical examination. The operational definition of FR starts from the “middle fingertip” as the reference point for measuring distance, however, it is defined as a calculated difference in the relative position of the shoulder joints on both sides using the depth information from the Kinect sensor. The assessment point on the shoulder could potentially be hindered or obstructed by the subject’s limb or body [55]. In addition, it results in overestimating the distance when the left joint is relatively backward compared to the right joint due to the “trunk rotation” movement. However, we found that there is no proportional bias in the achieved results as there is no regular relationship between the Kinect-based system and physical examination.

In this study, we adopted “manual timing” to calculate “walking speed” and compared it with Kinect data instead of adopting the motion analysis system (such as Vicon 3D motion analysis, Optotrak System 3D motion analysis, etc.) to provide huge data from the elderly population. The research investigations which employed the “manual timing” technique achieved correlations ranging from “moderate” to “high” levels [34, 56–58]. Similarly, the measurement results in gait analysis can be influenced by various methodological factors, such as the positioning of the Kinect sensor and the distance of the test conducted.

SUS presents the subject’s overall subjective feelings when using the system. The results showed that the subjects have an excellent overall user experience and indicate that the system is comprehensive and easy-to-use. Nevertheless, the percentage of people willing to use it frequently does not correspond to the percentage of the number of people who feel good about the system. According to the subjects’ feedback, some felt that the operation could be further simplified to flatten the learning curve. If it is placed at home, there are still some issues like space limitations or lack of motivation to conduct the test themselves. Based on the reverse questions, the subjects pointed out that they did not have the confidence to operate the fitness evaluation system independently. Those mentioned above are obstacles that reduce the willingness to use the system frequently. In addition, the relative lower facets in the QUIS are “the overall reflection to the operation screen” and “learning”. Some subjects respond that the font size is too small, the subtitles appear too fast, and the explanatory texts are too complicated to read for the elderly.

This research is one of the studies that rarely takes advantage of the Kinect system for the overall functional motion of the elderly. However, some limitations in the current study should be deliberated. While testing for specific actions remains subject to certain limitations, the determination of actions and processing of data can be enhanced by optimizing algorithms through mathematical adjustments. Techniques such as spline interpolation can be utilized to ensure the stability of sampling in the Kinect device. Additionally, the resolution of the depth image can be improved by employing algorithms like the randomized decision forest algorithm. These optimization measures contribute to overcoming challenges and improving the accuracy of action determination and data processing [30]. As for the method for Kinect to acquire motion information, we utilized the official SDK to conduct the skeleton tracking [59]. Considering that the functional reach and walking speed are only related to “moderate” in this study, we suggest that researchers can use the raw data as a base to adjust algorithms, or further explore other action parameters to establish a test with better validity. Furthermore, as indicated by the satisfaction results of this system among the elderly, the testers cannot personally operate the Kinect-based system with the current version of software. We aim at providing the user with training in “teaching sessions” with more intuitive gesture control features in the future. Based on the users’ feedback, it is also suggested that the “pre-training confirmation” is the key to amply the willingness of the elderly to use and enhance their acceptance of technology.

## Conclusions

COVID-19 has opened and popularized alternate forms of healthcare delivery, even in telehealth. In the overall research, inspecting functional motion with Kinect has moderate reliability and validity in functional reach and walking speed, while having good reliability and validity in the others. It indicates that if the “distance” between the tester and Kinect sensor changes during the test, it will cause errors in measurement. Even though Kinect as an inspection tool requires optimization of its algorithm and experimental settings, it still has the capability to capture and analyze motions with an easy-to-use feature. It also shows the potential to be widely used in clinical settings, and further be applied to inspect people who suffer from impaired motion function, such as stroke and asthenia, etc. Besides, it can also be eventually integrated into personalized training protocols [52, 60] based on the results of fitness evaluation, making it a great health assistant in telerehabilitation.

### Electronic supplementary material

Below is the link to the electronic supplementary material.

Supplementary Material 1

Supplementary Material 2

## Data Availability

The datasets used and/or analyzed during this study are available from the corresponding author on reasonable request.

## Abbreviations

AC: arm curl
CI: confidence interval
FR: functional reach
ICC: intraclass correlation coefficient
ICT: Information and communication technology
IoT: Internet of Things
LOA: limits of agreement
OLS: one-leg stance
QUIS: Questionnaire for User Interaction Satisfaction
STS: sit-to-stand
SUS: system usability scale
TMST: two-minute step test
TUG: timed-up to go

## References

[1] Organization WH. World report on ageing and health. World Health Organization; 2015.

[2] Hollander JE, Carr BG (2020). Virtually Perfect? Telemedicine for Covid-19. N Engl J Med.

[3] Portnoy J, Waller M, Elliott T (2020). Telemedicine in the era of COVID-19. J Allergy Clin Immunol Pract.

[4] Oliu-Barton M, Pradelski BSR, Woloszko N, Guetta-Jeanrenaud L, Aghion P, Artus P (2022). The effect of COVID certificates on vaccine uptake, health outcomes, and the economy. Nat Commun.

[5] Rostad HM, Skinner MS, Helleso R, Sogstad MKR (2020). Towards specialised and differentiated long-term care services: a cross-sectional study. BMC Health Serv Res.

[6] Liu L, Stroulia E, Nikolaidis I, Miguel-Cruz A, Rios Rincon A (2016). Smart homes and home health monitoring technologies for older adults: a systematic review. Int J Med Informatics.

[7] Anderson M, Perrin A (2017). Tech adoption climbs among older adults. Pew Res Cent.

[8] Stone RI, Gaugler JE, Kane RL (2015). Chapter 6 - factors affecting the future of Family Caregiving in the United States. Family Caregiving in the New Normal.

[9] Tomita MR, Mann WC, Stanton K, Tomita AD, Sundar V. Use of currently available Smart Home Technology by Frail Elders: process and outcomes. Top Geriatric Rehabilitation. 2007;23(1).

[10] Pedone C, Chiurco D, Scarlata S, Incalzi RA (2013). Efficacy of multiparametric telemonitoring on respiratory outcomes in elderly people with COPD: a randomized controlled trial. BMC Health Serv Res.

[11] Rifkin DE, Abdelmalek JA, Miracle CM, Low C, Barsotti R, Rios P (2013). Linking clinic and home: a randomized, controlled clinical effectiveness trial of real-time, wireless blood pressure monitoring for older patients with kidney disease and hypertension. Blood Press Monit.

[12] Gellis ZD, Kenaley BL, Have TT (2014). Integrated telehealth care for chronic illness and depression in geriatric home care patients: the Integrated Telehealth Education and activation of Mood (I-TEAM) study. J Am Geriatr Soc.

[13] Chen Z, Qi H, Wang L. Study on the types of Elderly Intelligent Health Management Technology and the influencing factors of its adoption. Healthc (Basel). 2021;9(11). 10.3390/healthcare9111494 PMC8619684 34828539

[14] Lepsy E, Radwanska E, Zurek G, Zurek A, Kaczorowska A, Radajewska A (2021). Association of physical fitness with quality of life in community-dwelling older adults aged 80 and over in Poland: a cross-sectional study. BMC Geriatr.

[15] Rikli RE, Jones CJ (1999). Development and validation of a functional fitness test for community-residing older adults. J Aging Phys Act.

[16] Zhao Y, Chung P-K (2016). Differences in functional fitness among older adults with and without risk of falling. Asian Nurs Res (Korean Soc Nurs Sci).

[17] Bethancourt HJ, Rosenberg DE, Beatty T, Arterburn DE (2014). Barriers to and facilitators of physical activity program use among older adults. Clin Med Res.

[18] Wilson J, Heinsch M, Betts D, Booth D, Kay-Lambkin F (2021). Barriers and facilitators to the use of e-health by older adults: a scoping review. BMC Public Health.

[19] Kosma M, Cardinal BJ, Rintala P (2002). Motivating individuals with disabilities to be physically active. Quest.

[20] Nikkhoo M, Niu C-C, Fu C-J, Lu M-L, Chen W-C, Lin Y-H (2021). Reliability and validity of a Mobile device for assessing Head Control ability. J Med Biol Eng.

[21] Rothman MD, Leo-Summers L, Gill TM (2008). Prognostic significance of potential Frailty Criteria. J Am Geriatr Soc.

[22] Ates HC, Nguyen PQ, Gonzalez-Macia L, Morales-Narvaez E, Guder F, Collins JJ (2022). End-to-end design of wearable sensors. Nat Rev Mater.

[23] Javaid M, Haleem A, Rab S, Pratap Singh R, Suman R (2021). Sensors for daily life: a review. Sens Int.

[24] Aurand AM, Dufour JS, Marras WS (2017). Accuracy map of an optical motion capture system with 42 or 21 cameras in a large measurement volume. J Biomech.

[25] Colyer SL, Evans M, Cosker DP, Salo AIT. A review of the evolution of Vision-Based motion analysis and the integration of Advanced Computer Vision Methods towards developing a Markerless System. Sports Med - Open. 2018;4(1). 10.1186/s40798-018-0139-y PMC5986692 29869300

[26] Mundher A, Jiaofei Z (2014). A real-time fall detection system in Elderly Care using Mobile Robot and Kinect Sensor. Int J Mater Mech Manuf.

[27] Guerra BMV, Ramat S, Gandolfi R, Beltrami G, Schmid M. Skeleton data pre-processing for human pose recognition using neural network. 2020:4265–8. 10.1109/EMBC44109.2020.9175588 33018938

[28] Choppin S, Wheat J (2013). The potential of the Microsoft Kinect in sports analysis and biomechanics. Sports Technol.

[29] Stamm O, Heimann-Steinert A (2020). Accuracy of Monocular two-dimensional pose estimation compared with a reference Standard for Kinematic Multiview Analysis: Validation Study. JMIR mHealth and uHealth.

[30] Clark RA, Bower KJ, Mentiplay BF, Paterson K, Pua Y-H (2013). Concurrent validity of the Microsoft Kinect for assessment of spatiotemporal gait variables. J Biomech.

[31] Clark RA, Pua Y-H, Fortin K, Ritchie C, Webster KE, Denehy L (2012). Validity of the Microsoft Kinect for assessment of postural control. Gait Posture.

[32] Clark RA, Pua Y-H, Oliveira CC, Bower KJ, Thilarajah S, McGaw R (2015). Reliability and concurrent validity of the Microsoft Xbox one Kinect for assessment of standing balance and postural control. Gait Posture.

[33] Baeza-Barragán MR, Labajos Manzanares MT, Ruiz Vergara C, Casuso-Holgado MJ, Martín-Valero R (2020). The use of virtual reality Technologies in the treatment of Duchenne muscular dystrophy: systematic review. JMIR mHealth and uHealth.

[34] Vernon S, Paterson K, Bower K, McGinley J, Miller K, Pua YH (2015). Quantifying individual components of the timed up and go using the kinect in people living with stroke. Neurorehabilit Neural Repair.

[35] Rikli RE, Jones CJ (2013). Development and validation of criterion-referenced clinically relevant fitness standards for maintaining physical independence in later years. Gerontologist.

[36] Clark RA, Pua YH, Fortin K, Ritchie C, Webster KE, Denehy L (2012). Validity of the Microsoft Kinect for assessment of postural control. Gait Posture.

[37] Michikawa T, Nishiwaki Y, Takebayashi T, Toyama Y (2009). One-leg standing test for elderly populations. J Orthop Sci.

[38] Duncan PW, Weiner DK, Chandler J, Studenski S (1990). Functional Reach: a New Clinical measure of balance. J Gerontol.

[39] Jones CJ, Rikli RE, Beam WC (1999). A 30-s chair-stand test as a measure of lower body strength in community-residing older adults. Res Q Exerc Sport.

[40] Shih M, Zhou J, Yang Y-R, Chen C (2019). Validation of an adaptive algorithm used in cost-effective Kinect-Based System for Gait Analysis. Arch Phys Med Rehabil.

[41] Brooke J (1996). SUS-A quick and dirty usability scale. Usability evaluation in industry.

[42] Bangor A, Kortum P, Miller J (2009). Determining what individual SUS scores mean: adding an adjective rating scale. J usability Stud.

[43] Koo TK, Li MY (2016). A Guideline of selecting and reporting Intraclass correlation coefficients for Reliability Research. J Chiropr Med.

[44] Barreira CC, Forner-Cordero A, Grangeiro PM, Moura RT (2020). Kinect v2 based system for gait assessment of children with cerebral palsy in rehabilitation settings. J Med Eng Technol.

[45] Panhwar YN, Naghdy F, Naghdy G, Stirling D, Potter J. Assessment of frailty: a survey of quantitative and clinical methods. BMC Biomedical Engineering. 2019;1(1). 10.1186/s42490-019-0007-y PMC7422496 32903310

[46] Yeung LF, Cheng KC, Fong CH, Lee WCC, Tong K-Y (2014). Evaluation of the Microsoft Kinect as a clinical assessment tool of body sway. Gait Posture.

[47] Eltoukhy MA, Kuenze C, Oh J, Signorile JF (2018). Validation of Static and Dynamic Balance Assessment using Microsoft Kinect for Young and Elderly populations. IEEE J Biomedical Health Inf.

[48] Wang S, Bolling K, Mao W, Reichstadt J, Jeste D, Kim H-C, et al. editors. Technology to support aging in place: older adults’ perspectives. Healthcare. Multidisciplinary Digital Publishing Institute; 2019. 10.3390/healthcare7020060 PMC6627975 30974780

[49] Mynatt ED, Rogers WA (2001). Developing technology to support the functional independence of older adults. Ageing Int.

[50] Damant J, Knapp M, Watters S, Freddolino P, Ellis M, King D (2013). The impact of ICT services on perceptions of the quality of life of older people. J Assist Technol.

[51] Akbari G, Nikkhoo M, Wang L, Chen CPC, Han DS, Lin YH et al. Frailty Level classification of the Community Elderly using Microsoft Kinect-Based Skeleton Pose: A Machine Learning Approach. Sensors. 2021;21(12). 10.3390/s21124017 PMC8230520 34200838

[52] Wochatz M, Tilgner N, Mueller S, Rabe S, Eichler S, John M (2019). Reliability and validity of the Kinect V2 for the assessment of lower extremity rehabilitation exercises. Gait Posture.

[53] Lencioni T, Carpinella I, Rabuffetti M, Cattaneo D, Ferrarin M. Measures of dynamic balance during level walking in healthy adult subjects: Relationship with age, anthropometry and spatio-temporal gait parameters. Proceedings of the Institution of Mechanical Engineers Part H, Journal of engineering in medicine. 2020;234(2):131 – 40. 10.1177/0954411919889237 31736408

[54] Eltoukhy MA, Kuenze C, Oh J, Signorile JF (2018). Validation of Static and Dynamic Balance Assessment using Microsoft Kinect for Young and Elderly populations. IEEE J biomedical health Inf.

[55] Leightley D, Yap MH, editors. Digital analysis of sit-to-stand in masters athletes, healthy old people, and young adults using a depth sensor. Multidisciplinary Digital Publishing Institute; 2018. Healthcare. 10.3390/healthcare6010021 PMC5872228 29498644

[56] Springer S, Yogev Seligmann G (2016). Validity of the Kinect for Gait Assessment: a focused review. Sensors.

[57] Clark RA, Vernon S, Mentiplay BF, Miller KJ, McGinley JL, Pua YH (2015). Instrumenting gait assessment using the Kinect in people living with stroke: reliability and association with balance tests. J Neuroeng Rehabil.

[58] Geerse DJ, Coolen BH, Roerdink M (2015). Kinematic validation of a multi-kinect v2 instrumented 10-Meter walkway for quantitative gait assessments. PLoS ONE.

[59] Criminisi A, Shotton J, Konukoglu E. Decision forests for classification, regression, density estimation, manifold learning and semi-supervised learning. Microsoft Research Cambridge, Tech Rep MSRTR-2011-114. 2011;5(6):12.

[60] Çubukçu B, Yüzgeç U, Zileli R, Zileli A (2020). Reliability and validity analyzes of Kinect V2 based measurement system for shoulder motions. Med Eng Phys.

