# Supplementary material for: Feasibility of a kinect-based system in assessing physical function of the elderly for home-based care
Source: BMC Geriatr. 2023 Aug 16;23:495. doi: 10.1186/s12877-023-04179-4 (PMC10429079; doi:10.1186/s12877-023-04179-4)
Supplement: Supplementary file 1 — Supplementary Material 1 [file 12877_2023_4179_MOESM1_ESM.doc]

## Appendix 1: System Usability Scale Questionnaire (© Digital Equipment Corporation, 1986.)

|  | **Strongly**  **Disagree 1** | **Strongly**  **Agree 5** |
| --- | --- | --- |
| I think that I would like to use this system frequently | |  |  |  |  |  | | --- | --- | --- | --- | --- | | |
| I found the system unnecessarily complex | |  |  |  |  |  | | --- | --- | --- | --- | --- | | |
| I thought the system was easy to use | |  |  |  |  |  | | --- | --- | --- | --- | --- | | |
| I think that I would need the support of a technical person to be able to use this system | |  |  |  |  |  | | --- | --- | --- | --- | --- | | |
| I found the various functions in this system were well integrated | |  |  |  |  |  | | --- | --- | --- | --- | --- | | |
| I thought there was too much inconsistency in this system | |  |  |  |  |  | | --- | --- | --- | --- | --- | | |
| I would imagine that most people would learn to use this system very quickly | |  |  |  |  |  | | --- | --- | --- | --- | --- | | |
| I found the system very cumbersome to use | |  |  |  |  |  | | --- | --- | --- | --- | --- | | |
| I felt very confident using the system | |  |  |  |  |  | | --- | --- | --- | --- | --- | | |
| I needed to learn a lot of things before I could get going with this system | |  |  |  |  |  | | --- | --- | --- | --- | --- | | |
