# Supplementary material for: Feasibility of a kinect-based system in assessing physical function of the elderly for home-based care
Source: BMC Geriatr. 2023 Aug 16;23:495. doi: 10.1186/s12877-023-04179-4 (PMC10429079; doi:10.1186/s12877-023-04179-4)
Supplement: Supplementary file 2 — Supplementary Material 2 [file 12877_2023_4179_MOESM2_ESM.doc]

## Appendix 2: Questionnaire for User Interface Satisfaction (Chin, J.P., Diehl, V.A., Norman, K.L., 1988)

Please rate your satisfaction with the system. *7-point Likert scale from 1 (strongly dissatisfied) to 7 (strongly satisfied)

1. Overall reaction to the Software:
   - Terrible / Wonderful
   - Difficult / Easy
   - Frustrating / Satisfying
   - Dull / Stimulating
   - Rigid / Flexible
2. Screen
   - Reading characters on the screen – Hard / Easy
   - Organization of information – Confusing / Very Clear
   - Sequence of screens – Confusing / Very Clear
3. Terminology and system information
   - Use of terms throughout system – Inconsistent / Consistent
   - Terminilogy is intuitive – Never / Always
   - Position of messages on screen – Inconsistent / Consistent
   - Prompts for input – Confusing / Clear
   - Error messages – Unhelpful / Helpful
4. Learning
   - Learning to operate the system – Difficult / Easy
   - Performing tasks is straightforward – Never / Always
